# Distribution and Seasonality of the Omura’s Whale (*Balaenoptera omurai*) in Australia Based on Passive Acoustic Recordings

**DOI:** 10.3390/ani14202944

**Published:** 2024-10-12

**Authors:** Ciara Edan Browne, Christine Erbe, Robert D. McCauley

**Affiliations:** Centre for Marine Science and Technology, Curtin University, Perth, WA 6102, Australia; c.erbe@curtin.edu.au (C.E.); r.mccauley@cmst.curtin.edu.au (R.D.M.)

**Keywords:** distribution, seasonality, Omura’s whale, passive acoustics

## Abstract

**Simple Summary:**

The Omura’s whale was first described in 2003 and is currently listed as ‘Data Deficient’ on the IUCN Red List. Distributed globally in tropical waters, there remains an absence of knowledge on the species’ behaviour and ecology, especially in Australian waters. This study utilised historic acoustic data to provide the first wide-scale investigation into Omura’s whale distribution and seasonality around Australia, including any potential migratory movements they undertake. We identified the species’ acoustic presence on the east coast of Australia for the first time and its seasonal presence on the west coast. Unlike most baleen whales which migrate between the poles and the equator annually with separated feeding and breeding grounds, the Omura’s whale inhabits certain regions around Australia for all months of the year. Currently, the Omura’s whale is not listed in any management or conservation policies in Australia so the description of when and where they are inhabiting will provide the first steps in developing these policies for the species in Australia, which is particularly important given that the identified distribution of the species crosses over with a lot of offshore industry operations.

**Abstract:**

The Omura’s whale (*Balaenoptera omurai*) is one of the most recently described species of baleen whale. Initially known only from stranding and whaling specimens, it has now been identified in all ocean basins excluding the central and eastern Pacific. Unlike most baleen whales that migrate between the poles and the equator seasonally, the Omura’s whale is known to inhabit tropical to sub-tropical waters year-round. In Australian waters, there remain fewer than 30 confirmed visual sightings over the past decade. However, based on acoustic records, the Omura’s whale has been detected off areas of the northwest coast of Australia year-round. This study utilises passive acoustic recordings from 41 locations around Australia from 2005 to 2023 to assess the distribution and seasonality of the Omura’s whale. The seasonal presence of Omura’s whale vocalisations varied by location, with higher presence at lower latitudes. Vocalisations were detected year-round in the Joseph Bonaparte Gulf in the Timor Sea, and near Browse Island and Scott Reef, in the Kimberley region. In the Pilbara region, acoustic presence mostly peaked from February to April and no acoustic presence was consistently observed from July to September across all sites. The most southerly occurrence of Omura’s whale vocalisations was recorded off the North West Cape in the Gascoyne region. Vocalisations similar but not identical to those of the Omura’s whale were detected in the Great Barrier Reef. The identified seasonal distribution provides valuable information to assess environmental and anthropogenic pressures on the Omura’s whale and to aid in creating management and conservation policies for the species in Australia.

## 1. Introduction

The Omura’s whale (*Balaenoptera omurai*) was described as a new species of baleen whale in 2003 [1]. The initial description of the species was based on a stranding specimen, the holotype, from the Sea of Japan in 1998 along with eight historic whaling specimens collected from the waters surrounding the Solomon and Cocos (Keeling) Islands in the 1970s [1]. Prior to the species description, sightings and vocalisations of the Omura’s whale were attributed to the Bryde’s whale (*B. edeni*), but as a small or pygmy form due to its distinctly smaller size at maturity [1,2]. The combination of morphological and genetic analyses of the nine specimens distinguished them as a single species, distinct from any of the known species of baleen whale [1,2,3].

Ten years after the species’ initial description, an Omura’s whale was identified alive in the wild for the first time off the northwest coast of Madagascar, where the first and currently only in situ study of the species has been undertaken [2,4]. The Madagascan field study provided detailed descriptions of the Omura’s whale’s appearance and behaviour, as well as the sounds it produces under water [2]. This new information has resulted in several confirmed reports of the species around the world [4]. The species is thought to have a predominately coastal distribution in tropical to sub-tropical waters [4]. The current known range of the Omura’s whale includes the Indo-Pacific, Indian, and North and South Atlantic Oceans [4]. Interestingly, there are no reports to date from the Central or Eastern Pacific (neither North nor South Pacific).

Generally, baleen whales undertake annual seasonal migrations between the poles and the equator. Summer months are spent feeding in productive, high-latitude waters and winter months in warmer, low-latitude waters for breeding and calving [5,6]. However, some species of baleen whale have been found to inhabit tropical to sub-tropical waters year-round, such as the Omura’s, Bryde’s and Rice’s (*B. ricei*) whales [4,6,7,8,9]. They may move towards and away from the equator or inshore and offshore but do not embark on large-scale migrations covering many tens of degrees of latitude [6]. The population of Omura’s whales studied off northwest Madagascar is understood to be a resident, non-migratory population. The acoustic presence of this population has been recorded in the area for all weeks of the year; satellite telemetry data of a few individuals demonstrated localised movements only within a restricted range; and Omura’s whales have been reported to both feed and calve in the same habitat areas [2,10]. In the Chagos Archipelago, near Diego Garcia, vocalisations attributed to Omura’s whales have been recorded during 11 months of the year, which are thought to be produced by a local population, distinct from the northwest Madagascan population, which resides in the area year-round [4,11,12]. Off northwest and northern Australia, Omura’s whale vocalisations have also been identified in all months of the year with some indication of small-scale migrations in these regions [13,14].

In Australian waters, the first records of Omura’s whales were the historic whaling specimens captured offshore from the Cocos (Keeling) Islands in the 1970s [1,4]. Off the mainland, the Omura’s whale was first documented in January 2000, after becoming stranded in the Gulf of St. Vincent, South Australia. This stranding remains the most southerly report of the species and is thought to be an anomalous record in temperate waters [4,15]. All other accounts of the species in Australia have been in tropical to sub-tropical waters, predominantly along the northwest coast from Exmouth to the Timor Sea, with a few sightings in recent years in the Great Barrier Reef [4,16,17]. Of the accounts across the northwest coast, there remain fewer than 30 confirmed visual sightings over the past decade. However, the Centre for Whale Research and Curtin University’s Centre for Marine Science and Technology have confirmed similar vocalisations to those described by Cerchio et al. [2] with concurrent visual observations of an Omura’s whale along the North West Shelf [4,16]. Northwest Australia is currently the only location other than northwest Madagascar where Omura’s whale vocalisations have been recorded with concurrent visual observations. These vocalisations have been detected in some areas of northwest Australia year-round [4,13,14].

Given the low number of sightings and large knowledge gaps on their current distribution, Omura’s whales are good subjects for passive acoustic monitoring (PAM). Through the detection of species’ stereotypical sounds, PAM can be used to determine the temporal presence of a species’ presence within a given area [18,19]. In comparison to visual monitoring methods, PAM is a cost-effective and non-invasive alternative which can provide long-term and 24-h sampling [19,20,21,22]. As it is not reliant on visibility, PAM is not impacted by daylight or visibility conditions and is less affected by weather conditions, making it suitable for use in unfavourable or remote environments where the opportunity for visual observations is limited [19,20,22,23,24]. However, true absences cannot be determined by PAM, as an animal could be present but not producing sound. For cryptic species, such as the Bryde’s and Rice’s whale, PAM has provided valuable information on spatial and temporal presence and migratory movements [7,25,26,27]. An understanding of the acoustic repertoire of the species of interest is, however, a prerequisite to the use of PAM techniques for investigating spatial and temporal presence.

The Omura’s whale produces a distinct, low-frequency (~15–60 Hz), amplitude-modulated (AM) vocalisation [2,16]. The vocalisation has been recorded with concurrent visual detections in both northwest Madagascar and northwest Australia [2,16]. In the Madagascan region, the vocalisation is between 15 and 50 Hz with a mean duration of 9.2 ± 0.92 s and repeated every 134.4–176.8 s [2]. In northwest Australia, the vocalisation is between 20 and 60 Hz, with a duration of 12–15 s and repeated every 160–210 s [16]. Similar vocalisations without visual confirmation have been attributed to Omura’s whales in the Chagos Archipelago (AM vocalisation, 17–50 Hz, duration 13.1 ± 0.6 s every 186 ± 18 s) and the Equatorial Atlantic Ocean (AM vocalisation, 20–47 Hz, duration 10.42 ± 1.16 s every 172.18 ± 32.5 s) [11,28]. Overlapping vocalisations from multiple individuals have been observed, indicating chorusing which is often thought to be a male breeding display [2,28,29]. The stereotyped sounds are rhythmically repeated, often at a consistent rate, and calling can continue for several hours to days [2,16,28,30]. These are traits of baleen whale song and Cerchio et al. [2] has suggested that Omura’s whale song could be a male-only trait [31,32,33]. In Australia, the vocalisation is mostly observed as a two-part sound or a ‘doublet’ whereas in other regions it can occur as a ‘singlet’ [2,16]. The second unit of the doublet vocalisation can have more tonal characteristics; however, the duration and frequency range of the vocalisations are similar across all regions. PAM is thus a promising method for studying Omura’s whale presence.

This study utilised long-term, archival PAM data to identify the acoustic presence of *B. omurai* around Australia. The aim was to define the distribution and seasonality, as well as any potential migratory patterns or movements of the Omura’s whale around the continent.

## 2. Methods

### 2.1. Data Collection

**Figure 1:**
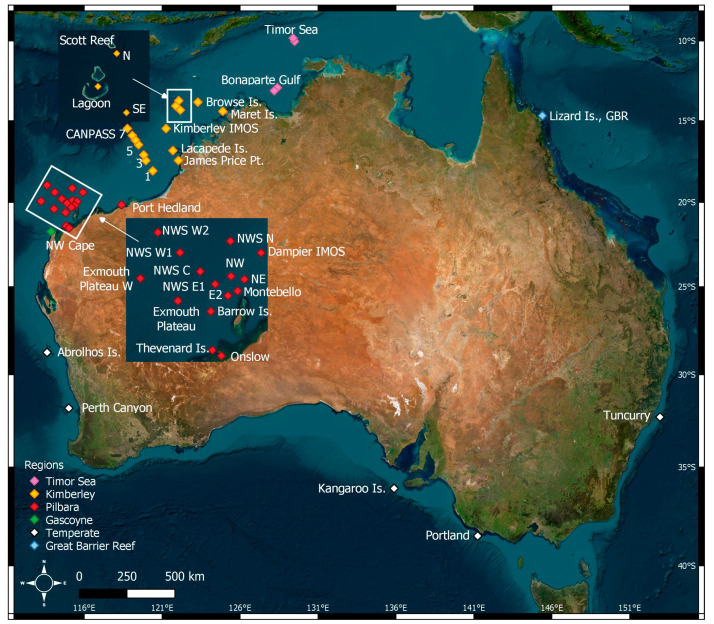
Deployment locations of USRs.

A total of 499,923 h of underwater acoustic recordings were collected from 41 offshore sites around Australia between 2005 and 2023. Regions where recordings were collected include the Timor Sea, Kimberley, Pilbara, Gascoyne, Abrolhos Islands and Perth Canyon in the Indian Ocean; off Kangaroo Island and Portland along the southern coast of Australia; and off Tuncurry and Lizard Island in the Pacific Ocean (Table 1, Figure 1). The CANPASS and North West Shelf (NWS) datasets in the Kimberley and Pilbara regions, respectively, were lateral sequences of recording instruments crossing the continental shelf from east to west. All recording instruments were moored to the seafloor at depths between 11 and 4162 m, except for two of the NWS C recording instruments, which were suspended in the water column at 200 m depth. Deployments lasted from 55 days to 436 days, with an average length of deployment of 220 days.

Five types of acoustic recorders were used to collect data (Table 1). Underwater acoustic recorders off the Kimberley (IMOS set), Dampier, the Perth Canyon, Kangaroo Island, Portland and Tuncurry were deployed as part of Australia’s Integrated Marine Observing System (IMOS). The CANPASS data was collected by Macquarie University and the Chinese Academy of Sciences, and all other datasets were collected by Curtin University’s Centre for Marine Science and Technology [34]. Most recordings were collected on the Centre for Marine Science and Technology and Defence Science and Technology Organisation underwater sound recorders (CMST-DSTO USRs) and were programmed to record 200 to 500 s samples every 900 s [35]. The CANPASS dataset was recorded on Gürlap ocean bottom seismometers (OBS; Güralp Systems Ltd., Reading, United Kingdom) from the Australian National Seismic Imaging Resource, which collected hour-long recordings every hour at 100 Hz [36]. Other acoustic recording instruments used were the SoundTrap 500STD (ST500; Ocean Instruments, Auckland, New Zealand), Song Meter Marine SM3M recorders (WLA; Wildlife Acoustics, Inc., Maynard, MA, USA) and LS1XL recorders (LH; Loggerhead Instruments Inc., Sarasota, FL, USA). All systems had a flat frequency response over the bandwidth of Omura’s whale calls. Sampling frequencies and duty cycles for each dataset are specified in Table 1 [37,38,39]. The USR, OBS and LH instruments were calibrated for system frequency response by inserting white noise of known spectral level in series with the hydrophone, whereas the ST500 and WLA instruments came manufacturer-calibrated.

### 2.2. Data Analysis

**Figure 2:**
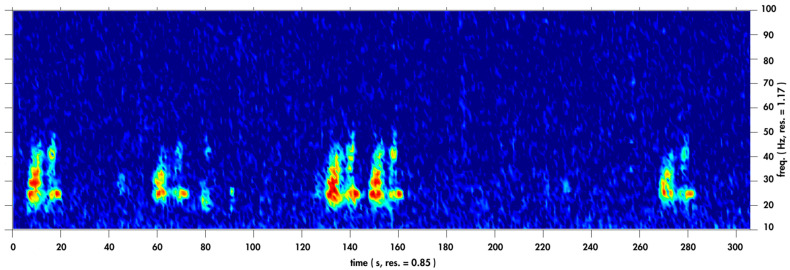
Example spectrogram of five Omura’s whale vocalisations detected off northwest Australia. Spectrogram produced using a 512-point Hanning window with 1.172 Hz and 0.85 s frequency and time resolution, respectively; sampling frequency 600 Hz.

Due to the large number of acoustic recordings, an automated acoustic detection algorithm was implemented in MATLAB (version 2019b; The MathWorks Inc., Natick, MA, USA) to detect the presence of Omura’s whale vocalisations. An example of an Omura’s whale doublet vocalisation is shown in Figure 2.

**Figure 3:**
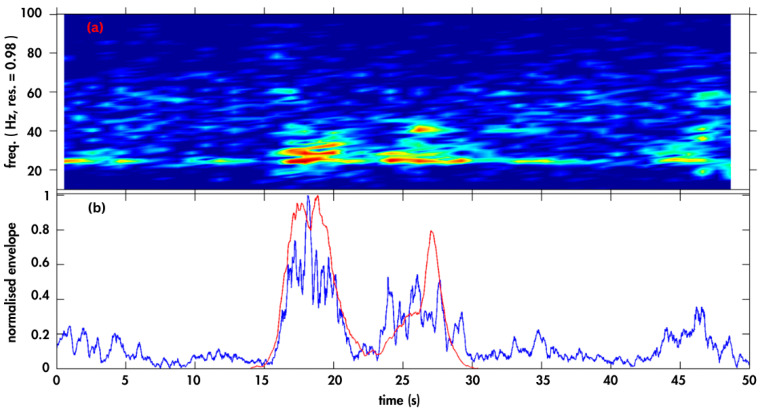
(**a**) Spectrogram of a section of a recording sample with an Omura’s whale vocalisation present at 15–30 s, and (**b**) normalised amplitude envelope of the sample (blue curve) compared to the Omura’s whale vocalisation template (red curve).

The detection algorithm looked for the characteristic waveform envelope shape of an Omura‘s vocalisation as displayed in Figure 3, then checked that the energy was in the correct frequency band and that surrounding energy (below and above the vocalisation frequency) was not high compared with in-band energy, indicating the vocalisation was not a noise artefact.

To do this, we carried out these processes: (1) loaded a sound file; (2) down-sampled to 250 Hz; (3) band-pass filtered the recording between 5 and 100 Hz using a fifth-order Butterworth filter; (4) calculated the magnitude of the Hilbert transform (envelope) and smoothed this (125-point running linear fit); (5) split the envelope over the whole file into 16.384 s sections each centred 1.636 s apart; (6) normalised each envelope section; (7) calculated the cumulative least-squares error between the normalised section and a normalised Omura’s vocalisation template envelope; (8) inverted this error value and reported a detection if the value was >20; (9) calculated the mean noise level across the adjacent (noise) frequency bands 10–15 Hz and 60–100 Hz over the section length; and (10) kept the detection when the noise level was 3 dB less than the Omura’s vocalisation level in the frequency band 18–50 Hz.

Locating Omura’s whale vocalisations in a dataset was a three-step process that included the following: (1) running the detection algorithm to identify the start point of each vocalisation within a sample; (2) manually checking all samples output by the detector and verifying, removing or adding vocalisation start points within a sample as appropriate; and (3) bracketing each verified sample with vocalisations by ±3 samples, adding missed vocalisation start points where appropriate and iterating this process until all samples with vocalisations had been checked for ±3 samples and no new vocalisations found within adjacent samples. Steps (2) and (3) were carried out such that only samples not previously checked were displayed for vocalisation verification. In terms of vocalisation detection, the detector performance varied across sites and datasets, with correct detections ranging from 67 to 100%, false detections from 1% to 9%, and missed detections from 27 to 95%. The manual checking process and its subsequent iterative sample bracketing ensured false and missed detections were accounted for so final vocalisation detections were validated. Despite the detection algorithm primarily being designed to detect the more common doublet vocalisation, it also detected singlet vocalisations, which was observed during the manual validation stage as almost every sample ends up being viewed. The seasonality of the two different call types was outside the scope of this study. The recurring presence of the two call types, especially in certain regions, was noted in the results, as it could be an area of further research.

To account for the different duty cycles across datasets, the timeframe of interest was the presence or absence of vocalisations per hour (Vhr). To determine the seasonal presence of vocalisations across sites, hours with vocal presence were divided by the total recorded hours per month to get a percentage of presence per month at each site (Vhr/month). For sites with multiple years of data, the data were grouped to show seasonality at the site over a 12-month period.

## 3. Results

Omura’s whale vocalisations were identified at 32 of the 41 recording sites around Australia, shown in Table 2. The seasonal presence of the vocalisations varied by location with some sites having a year-round presence. Vocalisations were detected in all months of the year at the Bonaparte Gulf, Browse Island, Scott Reef SE and Kimberley IMOS sites. There were no vocal detections of Omura’s whales at the Scott Reef lagoon, Port Hedland, Onslow, Exmouth Plateau, Abrolhos Island, Perth Canyon, Kangaroo Island, Portland and Tuncurry sites, for the years with data available. The most southerly detection of an Omura’s whale vocalisation was at the North West Cape site (21°45.08′ S, 113°52.71′ E), whereas the most northerly was in the Timor Sea (9°49.01′ S, 129°24.287’ E). The most westerly detection was at the NWS W2 site (18°57.51′ S, 113°37.14′ E) and on the East coast of Australia, sounds similar but not identical to those known to be produced by *B. omurai* were detected off Lizard Island.

### 3.1. Timor Sea Region

**Figure 4:**
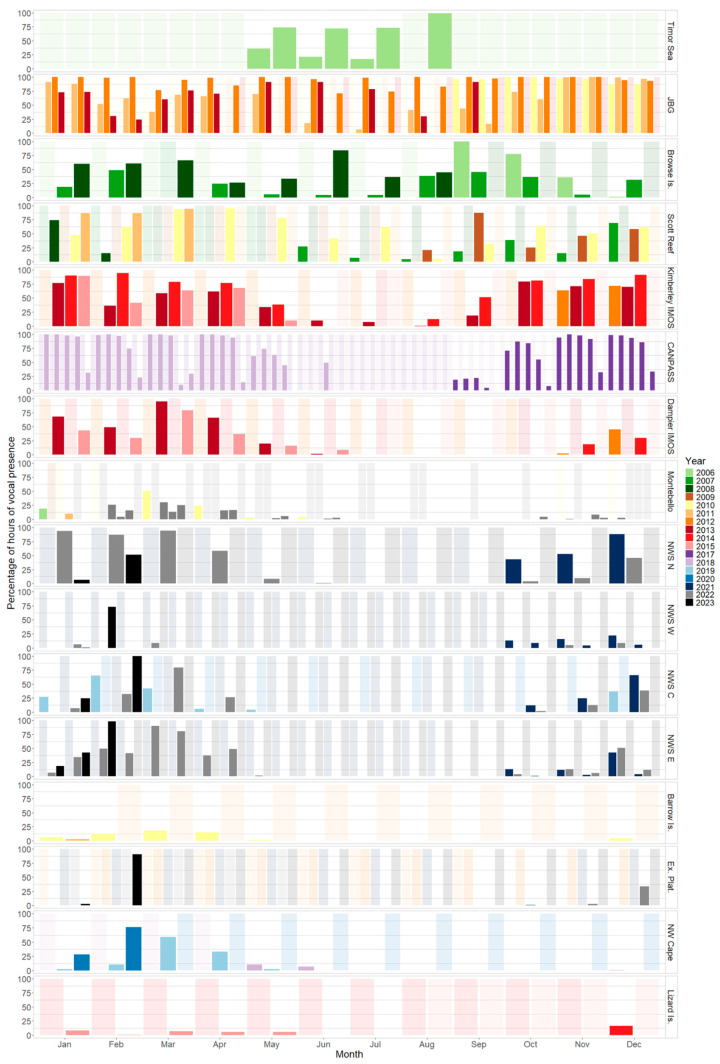
Bar plot of percentage of monthly vocal presence per recorded hours by site and year. Faded columns represent months with no data available. Sites with vocalisations in less than 15% of recordings were not included in this figure. JBG is the combined Bonaparte Gulf NE and SW sites. Scott Reef is the combined Scott Reef N and SE sites. CANPASS is the combined 1–7 sites in chronological order. Montebello is the combined Montebello, Montebello NW and NE sites. NWS W is the combined NWS W1 and W2 sites. NWS E is the combined NWS E1 and E2 sites. Ex. Plat. is the combined Exmouth Plateau and Exmouth Plateau W sites.

At the Timor Sea sites, there were only four months of data available from two USRs (Figure 4). Omura’s whale vocalisations were detected in all months with recordings from the first day of recording in late May until the last day of recording at the end of August. Both singlet and doublet vocalisations were detected in all months. In the Joseph Bonaparte Gulf (JBG), Omura’s whale vocalisations were observed year-round at both USR sites. The JBG sites had the highest %Vhr/month, which was greater than 61% for all months (Table 2). Vocal presence peaked from October to January, with %Vhr/month averaging 99.2% in November, then a shorter peak in April–May (Table 2). Both doublet and singlet vocalisations were observed on the JBG USRs.

### 3.2. Kimberley Region

**Figure 5:**
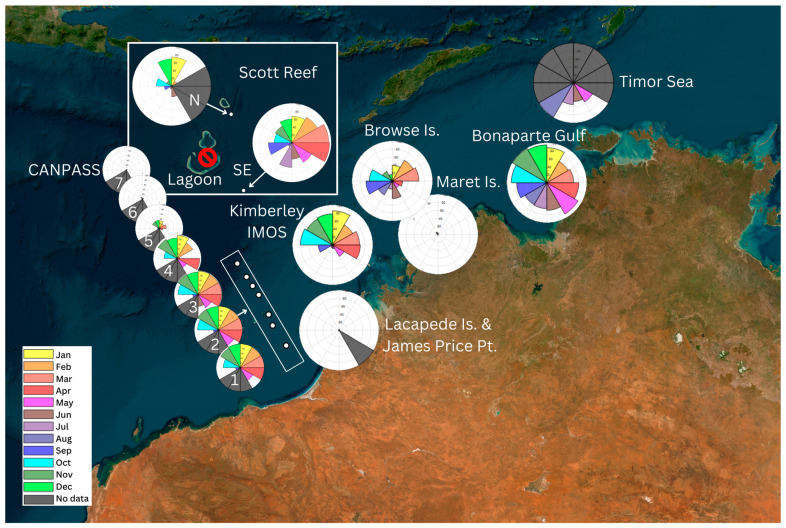
Map of seasonal distribution of Omura’s whale vocal presence in the Kimberley and Timor Sea regions. Polar histograms represent the percentage of recorded hours with Omura’s whale vocal presence per month whereas the yellow segment represents January, and months follow in a clockwise direction to December in bright green. The segments in black represent months with no acoustic data available. White segments correspond to recordings without Omura’s detections. The red slashed circles represent sites with no Omura’s whale vocal presence found in available data.

In the Kimberley region, vocal presence was detected in all months of the year at the Browse Island site (Figure 5). Vocal presence peaked in February–March and September–October (Vhr/month > 59%; Table 2). The Scott Reef SE site had a vocal presence in all months of the year with a peak in March and April (Vhr/month > 93%; Table 2). The Scott Reef N site had vocalisations present from June to February with a peak from December to January (Vhr/month > 69%) and no data available for March–May (Figure 5). No vocal detections were observed on the Scott Reef Lagoon USR for months with data available. The Maret Islands site had Omura’s whale vocalisations from November to May (Figure 5). Vocal presence was highest in November and December with 6.55% and 3.97% Vhr/month, respectively, while from January to May, Vhr/month < 1% (Table 2). The Kimberley IMOS site had a year-round vocal presence with peaks from October to January (Vhr/month > 75%) and in March–April (Vhr/month > 67%; Figure 5). From June to August, Vhr/month < 10% (Table 2). Near Broome, the Lacepede Islands USR had a low presence of vocalisations from November to April (Vhr/month < 2%) with no data available for May–July. The James Price point USR only had vocalisations in August for 0.27% of recording hours with no data available for January–May (Table 2).

At the CANPASS lateral sequence of OBSs, no data were available from July or August. Both singlet and doublet vocalisations were observed on the CANPASS USRs, the singlet vocalisations were mostly observed in March on the western USRs. The first three USRs (1–3), closest to the coast, detected a similar pattern of vocal presence (Figure 4; Figure 5). Vocalisations were present in over 93% of recording hours from November to April, over 61% in May and October, and ~20% in September (Table 2). Only the CANPASS 3 recorder had data from June (49% Vhr/month; Figure 4). For the remaining four USRs, Omura’s whale vocal presence decreased the farther away from the coast the USRs were located. The CANPASS 4 recorder had a presence from September to May, with peak Vhr/month from November to February and in April, but 10% Vhr/month in March (Table 2). The CANPASS 5 recorder had vocal presence from October to April (Vhr/month < 34%) and no vocal detections in May or September (Table 2). CANPASS 6 had vocal presence from November to March (Vhr/month < 10%) and only singlet vocalisations were detected in March (Table 2). CANPASS 7 only had a vocal presence for 2 days in March (3% Vhr/month) and only singlet vocalisations were detected (Table 2).

### 3.3. Pilbara Region

**Figure 6:**
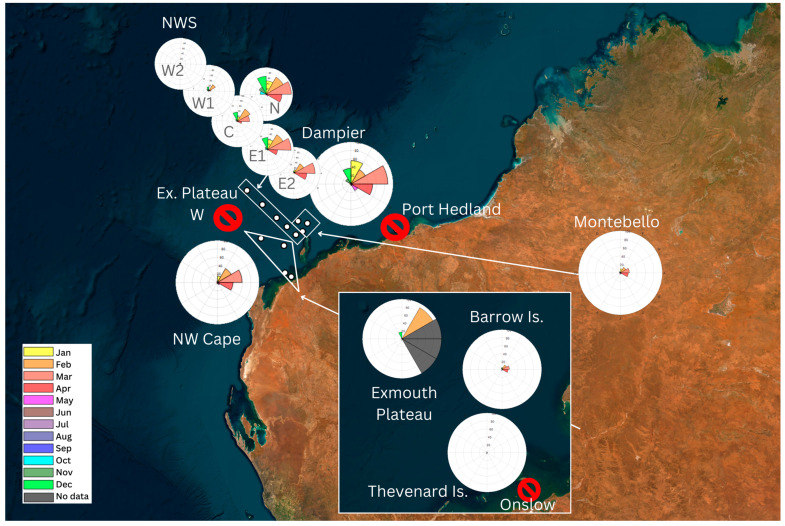
Map of seasonal distribution of Omura’s whale vocal presence in the Pilbara and Gascoyne regions. Polar histograms represent the percentage of recorded hours with Omura’s whale vocal presence per month, while the yellow segment represents January and months following in a clockwise direction to December in bright green. The segments in black represent months with no acoustic data available. White segments correspond to recordings without Omura’s detections. The red slashed circles represent sites with no Omura’s whale vocal presence found in available data.

In the Pilbara region, vocal absence was determined from July to September across all sites and presence mostly peaked from February to April (Figure 6). The Dampier IMOS site had a vocal presence from late October until mid-June. Vhr/month was highest in March (89%) and less than 5% in June and October (Table 2; Figure 4). The NWS N site had Omura’s whale vocal presence from October to June, with a peak Vhr/month in March (94.1%). At the combined Montebello sites, vocalisations were present from October to June with a peak from February to April and no presence in July–September (Table 2). The Barrow Island site had a vocal presence from December to May with a peak in February–April (Table 2). NWS E2 had a presence from October to June with peak Vhr/month from February to April and the highest presence in March (80.4%; Table 2). NWS E1 had vocal presence from October to May, while Vhr/month was greater than 37% from December to April and highest in March (90.2%; Table 2). NWS C had vocal presence from October to May with peaks in February–March and December (Figure 6). NWS W1 had a vocal presence from October to March, and Vhr/month was highest in February (28%) and December (15.2%; Table 2). NWS W2 site only had Omura’s whale vocal presence in October–December (Vhr/month < 4.5%; Table 2). Exmouth Plateau W had no vocal detections for years with data available. There was no data for September and ambient noise was frequently observed within the frequency range of Omura’s whale vocalisations. The Exmouth Plateau site had a vocal presence from October to February with no data available from March to May. Presence peaked in February with vocalisation detected in 90.3% of recordings (Table 2). The Thevenard Island site, which was near shore (<40 km from shore) had a vocal presence in March and April for less than 1.2% of recorded hours per month (Table 2). The Port Hedland and Onslow sites were both near shore (<25 km from shore) and vocalisations were not detected for years with data available. Port Hedland had no data available for the month of October (Table 2).

### 3.4. Gascoyne Region

The North West Cape site in the Gascoyne region had a vocal presence from December to June with a peak from February to April (Table 2; Figure 4). This site was the most southerly range of Omura’s whale vocalisations detected in this study (Figure 6).

### 3.5. Temperate Regions

No Omura’s whales were acoustically detected at the Abrolhos Island, Perth Canyon, Kangaroo Island, Portland and Tuncurry sites, for the years with data available (Table 2).

### 3.6. Great Barrier Reef

**Figure 7:**
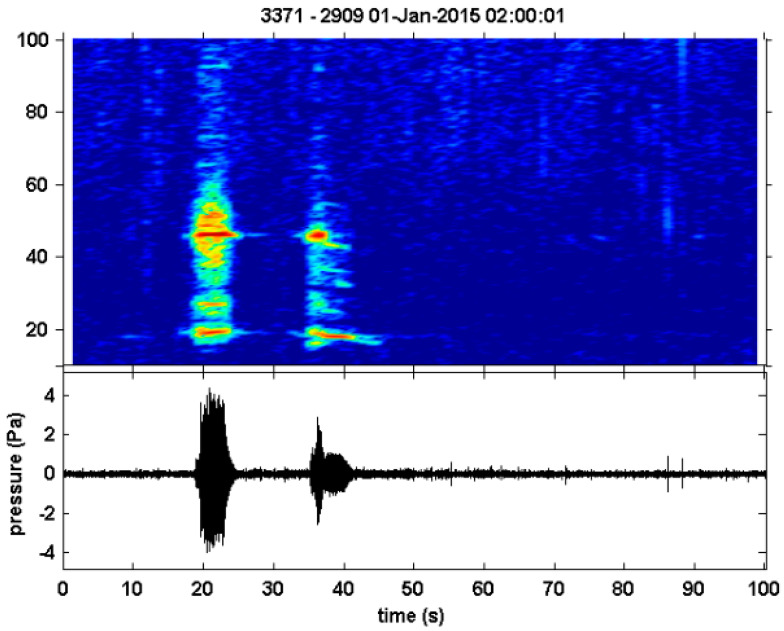
Sound spectrogram and waveform of potential Omura’s whale vocalisation detected off Lizard Island, Great Barrier Reef.

The USR off Lizard Island recorded sounds that were similar but not identical to those known to be produced by Omura’s whales. These 15–60 Hz AM sounds were detected by the automated detection algorithm and had the ‘doublet’ form (Figure 7). The sounds were present from December to May with the highest Vhr/month in December and no recordings available from August to November (Table 2; Figure 4). The sounds with a signal-to-noise ratio greater than 10 were measured to find an average duration of 13.87 ± 0.37 s and an average peak frequency of 24.92 ± 0.57 Hz. Due to the short sample length (180 s), the interval between calls could not be calculated accurately.

## 4. Discussion

Omura’s whale vocalisations were recorded in 32 out of 41 PAM sites sampled around Australia. The seasonality of Omura’s whale vocal presence varied by location, with a strong latitudinal pattern of distribution. The species had higher vocal presence detected at lower latitudes with year-round presence recorded in the Joseph Bonaparte Gulf and Kimberley region. Omura’s whale vocal presence mostly peaked during austral summer (December to February) and autumn (March to May) across all regions. In the Pilbara and Gascoyne regions, vocal presence was not observed during austral winter (June–August) and early spring (September). The most southerly detection of an Omura’s whale vocalisation was off the North West Cape, Western Australia, and no vocalisations were detected in temperate waters. Vocalisations that were similar (Figure 7) but not identical to those currently known of the Omura’s whale were detected in the Great Barrier Reef for the first time.

### 4.1. Spatial Patterns of Presence

The Omura’s whale is thought to be a non-migratory baleen whale species which remains in the tropics to sub-tropics year-round. All Omura’s whale vocalisations were recorded in tropical to sub-tropical waters in this study, consistent with the current known range of the species [4]. Vocal presence mostly peaked during austral summer and autumn across all regions. At the Bonaparte Gulf, Browse Island, Scott Reef SE and Kimberley IMOS sites, vocalisations were detected in all months of the year. As the recordings used in this study were archival, annual seasonal presence could not always be captured due to data incompleteness at some sites. At sites that did not have a year-round vocal presence, vocalisations were absent during austral winter and early spring. Vocalisations were absent in the Pilbara and Gascoyne regions from July to August, and had a low presence (Vhr/month < 10%) in May to June and September other than at the Dampier IMOS and NWS N sites. It is important to note that while the presence of Omura’s whale vocalisations indicates the presence of the species in the area, the absence of vocalisations may not mean an absence of the species as animals can be present and not vocalising. Currently, we do not know the function of Omura’s whale vocalisation and whether or not it is a male-only trait or associated with different behaviours or seasons. As further research is undertaken on the species, the distribution outlined in this study may be expanded further or allocated to males only.

There were no clear indications of migrations being undertaken by Omura’s whales in Australia. In general, the peaks in presence overlapped across regions, the Timor Sea region had high levels of vocal presence year-round, the Kimberley region had high presence from November to May, and the Pilbara region generally had the highest presence in February–April. The Pilbara region had no vocal detections from July to September, so we can presume Omura’s whales were not inhabiting the area during that time and moving elsewhere. However, even sites with year-round presence had lower vocal presence in the months of July–September and we cannot determine where they were moving based on the data in this study alone. The only exception to a low presence in August was the Timor Sea site, which had a vocal presence in 99% of recorded hours for that month. Presence in the month of August then decreased as site latitude increased. The Bonaparte Gulf site had a vocal presence in 83% of recorded hours and then the rest of the study sites had a vocal presence in less than 40% of recorded hours for the month of August. There were also no clear east to west or inshore to offshore movements along the west coast. The two lateral sequences, in the Kimberley (CANPASS 1–7) and Pilbara regions (NWS E2–W2), had a lower vocal presence on the USRs that were farthest offshore but the monthly trends in vocal presence were consistent east to west, e.g., there was only presence in March on the most western CANPASS USR, but on the most eastern USR, vocalisations were present for 100% of recorded hours in March. McPherson et al. [13] suggested Omura’s whales were moving east to west in the Timor Sea and exiting an area northeast of the Bonaparte Gulf site in a southwest direction around November. Omura’s whale vocal presence peaked at the Bonaparte Gulf site from October to January, so it is likely some Omura’s whales are moving northeast to southwest and back across the Timor Sea region throughout the year as suggested, while it is possible some stay closer to the Bonaparte Gulf site year-round.

Given that Omura’s whale vocalisations were detected year-round in parts of Australia, they are likely to be feeding and breeding within the same habitat areas, like the Omura’s whale population off northwest Madagascar [2]. There have been separate sightings of an Omura’s whale feeding and of an adult-and-calf pair as well as stranding of a sub-adult off the Ningaloo coast, so there are varying age demographics of the species and feeding opportunities in the same region [40,41]. Furthermore, pygmy blue (*B. musculus brevicauda*) and fin (*B. physalus*) whales have also been sighted feeding along the Ningaloo coast, and the Scott Reef area has been identified as a possible foraging area for pygmy blue whales with reliable prey sources available in the wider region for baleen whales [42,43,44,45,46,47]. Currently, it is not known what the Omura’s whale feeds in Australian waters. Field studies would be required to confirm if Omura’s whales are in fact feeding and breeding within the same habitats or if they use distinct habitats across the tropical waters of Australia to undertake these events.

### 4.2. Zero-Presence Sites

Despite years of archival recordings, there was no Omura’s whale vocal presence in temperate waters, which included the Abrolhos Island, Perth Canyon, Kangaroo Island, Portland and Tuncurry sites. The lack of vocal presence along the southern continental shelf reiterates that the Omura’s whale is a tropical species and that the stranding in the Gulf of St. Vincent, the only report of the species along the southern coast of Australia, was an anomalous record. Some sites within tropical waters had zero vocal presence detected, including the Scott Reef lagoon, Onslow, Exmouth Plateau West and Port Hedland sites. There were also tropical sites with near zero presence (Vhr/month < 10%), including Maret Islands, James Price Point, Lacepede Island, CANPASS 6 and 7, Thevenard Island and NWS W2.

The Scott Reef lagoon USR was located in between the two southerly atolls of Scott Reef in 51 m of water. Vocalisations were detected on the other USRs around Scott Reef, all of which were located at over 300 m depth. The lack of vocal presence at this site suggests Omura’s whales are unlikely to be entering the southern lagoon of Scott Reef, at least in the months with data available, and instead inhabiting the surrounding waters. This has also been observed in pygmy blue whales, which do not appear to enter or vocalise in the lagoon either [14]. The Onslow, Port Hedland, Thevenard Island, Maret Islands and James Price Point sites were all in less than 50 m of water and within 30 km of the coastline. The Lacepede Islands site was within 70 km of the coast and within 42 km of islands in 31 m of water. There was an Omura’s whale sighting north of the Lacepede Islands (122°09.36′ E) on the 2nd of November 2009 but no vocalisations were present in the recordings for that day [48]. The exact time and location of the sighting were not given but the animal was either vocalising outside the USR’s detection range or duty cycle or not vocalising at all. These locations with minimal or no presence are potentially too shallow or too close to the coastline or land masses to be regular habitats for the species in this region, whereas, the Exmouth Plateau West, NWS W2, and CANPASS 6 and 7 sites were potentially too deep or offshore to be regular habitats for the species, although they have been reported in deep water elsewhere [4,11,28]. The depths of the USRs will also impact acoustic propagation and detection range of the vocalisations [22,49,50,51].

### 4.3. Detection Range

The detection ranges of the USRs vary with site and depend on bathymetry, bottom topography and composition, and oceanographic variables [49]. The marine soundscape can have ambient noise sources from geophony (e.g., wind, rain and breaking waves), biophony (e.g., marine mammals, fishes and crustaceans), and anthropophony (e.g., ships, boats and seismic surveys), which can affect the signal-to-noise ratio of vocalisations and thus the detection range [52,53]. High levels of ambient noise were noted at the Exmouth Plateau West site within the same frequency band as the Omura’s whale vocalisation but no vocalisations were detected. The lack of vocal presence could be due to the absence of vocalisations or limited detectability due to ambient noise. The detection range might change with season, due to variable hydroacoustic water parameters (e.g., temperature profile). Detection range variability might thus confound some of the vocal presence percentages; however, by selecting a minimum 3 dB signal-to-noise ratio, weak and potentially farther-away vocalisations were excluded. (We say ‘potentially farther away’ as the noise level had been computed over the same time window as the signal level, but outside of the signal band, and so, in-band ambient noise could still have affected detection range variability.) Differing grades of the vocalisations detected can indicate that the sounds originated from different ranges, were affected by noise, or were emitted at variable source level [11]. Currently, the source level of the Omura’s whale is unknown; so, we cannot determine the ranges of the vocalising animals. Given that Omura’s whales produce low-frequency sounds, like other baleen species, it is likely they can be detected from several tens of kilometres, depending on the environment [12,25,28].

Omura’s whales likely vocalise within the top 40 m of water, as other baleen whales do [54,55,56,57]. In certain circumstances, their vocalisations might propagate very far. If the whales vocalise over the continental slope, then their vocalisations might scatter into the deep sound channel with one bottom bounce. Once in the sound channel (which has an axis at 1000 m depth in low latitudes), sound propagates very well without sea surface or seafloor interactions and thus losses. USRs inside the channel (as those at the NWS sites) might thus pick up vocalisations from as far away as the continental slope. It would be useful to deploy a hydrophone array in the future, to localise the vocalisations, determine their source level, and compute the detection ranges [35,58].

### 4.4. Depth Range

Off Madagascar, Omura’s whales were encountered in waters from 4 to 202 m but predominantly in shallow waters (10–25 m); in the Equatorial Atlantic, they were detected on hydrophones deployed at 700 m in 3000 m of water; and Omura’s whales have been sighted feeding off Indonesia in 60–180 m depth [2,4,28]. In this study, Omura’s whale vocalisations were detected on USRs in water depths of 21 m to 4162 m. However, there was minimal presence at these two extremes. The mean depth of USR sites with detections was 507.4 m ± 689.3 m and the mean depth of sites with year-round presence was 168.6 m ± 90.1 m. As previously mentioned, the detection range will differ at each site and the depth of the USRs is not directly indicative of the depth of the habitat animals are inhabiting. In general, most detections were from the continental shelf with some deeper-water detections. This distribution pattern is similar to where the species has been reported globally [4].

### 4.5. Singlet vs. Doublet Vocalisation

The ‘doublet’ vocalisation was present at all sites with vocal presence except for the CANPASS 7 USR, the most westerly in the CANPASS sequence, which only had ‘singlet’ type vocalisations. Both vocalisation types were detected in all months with data available in the Timor Sea and Bonaparte Gulf datasets and often within the same sample. Elsewhere, the ‘singlet’ vocalisation was only detected in a few samples to a few months per dataset and the most southerly detection of the ‘singlet’ vocalisation was at the NWS E2 site. Singlet vocalisations were detected at the CANPASS 6 and 7 sites for only 2 days in March but were not detected on any of the other CANPASS recorders. The variation in vocalisation type suggests there may be different acoustic populations of Omura’s whales around Australia or the species may have different call types associated with different behaviours [12,31,32]. Further research of acoustic data with genetic observations would be needed to determine this.

### 4.6. Great Barrier Reef Detections

The sound detected off Lizard Island was likely produced by a baleen whale due to its low-frequency range and duration. However, without concurrent visuals, the sound source cannot be confirmed. Of the known baleen whale vocalisations, the sound is most similar to the Omura’s whale vocalisation given it is comprised of two parts, the first being amplitude-modulated between 15–60 Hz. The automated detection algorithm designed specifically for Omura’s whale vocalisations used in this study also detected this sound type as an Omura’s whale vocalisation due to the similarities in frequency, amplitude envelope and duration. The sounds had a similar average duration (13.87 ± 0.37 s) compared to Omura’s whale vocalisations detected off northwest Australia (12–15 s)and the Chagos Archipelago (13.1 ± 0.6 s) [11,16]. However, they were longer than those detected off Madagascar (9.2 ± 0.92 s) and in the Equatorial Atlantic Ocean (10.42 ± 1.16 s) [2,28]. The average peak frequency (24.92 ± 0.57 Hz) was lower than that of Omura’s whale vocalisations observed off Madagascar (36.1 ± 6.19 Hz) [2], Furthermore, the sound had the highest presence in December and most of the Omura’s whale sightings on the Great Barrier Reef have been during late November and December, only some of which have been confirmed sightings [4,59,60]. The Lizard Island sound is likely another acoustic population of the species in Australia and the first time Omura’s whales have been acoustically detected off the east coast of Australia. Further acoustic data from the region and sightings alongside acoustics would be needed to confirm this.

### 4.7. Future Directions

Continuation of PAM will be useful to monitor trends in species presence long-term and could be used to identify areas for further field studies as have been conducted off Madagascar. Investigation into the species’ call characteristics and patterns in Australia to help us understand the function of the Omura’s whale vocalisation, its associated behaviours, and whether it is presented as a song will further our understanding of the species’ ecology and behaviour. Genetic sampling alongside acoustics could be used to identify whether the observed geographic variation in vocalisations indicates there are small localised populations around Australia as have been identified off Madagascar. Utilising a hydrophone array to localise Omura’s whale vocalisations, and determine their source level and detection ranges would further our understanding of where the species inhabits. Additionally, investigating the environmental variability of the sites Omura’s whales inhabit could identify drivers of the species distribution.

## 5. Conclusions

Currently, the Omura’s whale is listed as Data Deficient on the International Union for Conservation of Nature (IUCN) Red List [61]. In Australia, the species was only listed under the Environmental Protection and Biodiversity Conservation Act 1999 in September 2024 [62]. Identifying the seasonality and distribution of the Omura’s whale in Australia is crucial for furthering our understanding of the species’ ecology and assessing the species’ exposure to potential pressures, which can then inform environmental impact assessment and management. In particular, the current and expanding offshore oil and gas sector along Australia’s northwest coast significantly overlaps with Omura’s whale distribution identified in this study [63]. Underwater noise from offshore industry can interfere with key life processes of marine mammals by masking acoustic communication, impacting hearing, or changing behaviour [64,65,66]. The Omura’s whale also does not appear to migrate like most baleen whale species and instead inhabits the tropical to sub-tropical waters around parts of Australia year-round. Typically, the offshore resource industry in Australia undertakes operations outside of biologically important areas (e.g., breeding or foraging grounds) at biologically important times (e.g., peak migration) to minimise the noise exposure and impacts on marine mammal species of interest [67]. Given that the Omura’s whale is present for all months of the year, the species is put at higher risk of exposure to anthropogenic noise pollution from periodic seismic surveys and local, fixed installations.

## Figures and Tables

**Table 1:** Details of underwater acoustic recorder deployments. Duty cycle refers to mean sample length/mean sample increment (in seconds).

Location	Latitude	Longitude	Start Date	End Date	Effort [h]	Recorder Depth [m]	Duty Cycle [s/s]	Acoustic Recorder Type	Sampling Frequency [kHz]
Timor Sea region									
Timor Sea	10°01.33′	129°29.98′	25/05/2006	17/07/2006	1272	116	500/900	CMST-DTSO USR	6
	9°49.01′	129°24.29′	25/05/2006	29/08/2006	2304	281	400/900	CMST-DTSO USR	8
Bonaparte Gulf NE	12°56.04′	128°23.86′	15/09/2010	9/03/2011	4197.5	96	300/900	CMST-DTSO USR	6
	12°56.06′	128°23.85′	10/09/2011	31/03/2012	4863.75	96	300/900	CMST-DTSO USR	6
	12°56.04′	128°23.85′	2/04/2012	19/09/2012	4082	96	300/900	CMST-DTSO USR	6
	12°55.97′	128°23.85′	20/09/2012	19/03/2013	4332.5	96	300/900	CMST-DTSO USR	6
Bonaparte Gulf SW	13°07.78′	128°11.48′	16/09/2010	9/03/2011	4177.75	95	300/900	CMST-DTSO USR	6
	13°07.74′	128°11.52′	9/03/2011	18/09/2011	4627	95	300/900	CMST-DTSO USR	6
	13°07.78′	128°11.49′	18/09/2011	31/03/2012	4679.25	95	300/900	CMST-DTSO USR	6
	13°07.78′	128°11.47′	1/04/2012	19/09/2012	4103.75	95	300/900	CMST-DTSO USR	6
	13°07.70′	128°11.39′	20/09/2012	20/03/2013	4350.25	95	300/900	CMST-DTSO USR	6
	13°07.80′	128°11.48′	22/03/2013	14/09/2013	4221.25	95	300/900	CMST-DTSO USR	6
Kimberley region									
Browse Is.	13°50.44′	123°17.63′	13/09/2006	3/02/2007	3435	247	300/900	CMST-DTSO USR	6
	13°50.31′	123°17.71′	1/04/2007	12/11/2007	5403.75	246	200/900	CMST-DTSO USR	6
	13°50.30′	123°17.83′	30/11/2007	11/08/2008	6123	246	200/900	CMST-DTSO USR	6
Scott Reef N	13°45.01′	122°04.00′	14/06/2007	4/02/2008	5630	363	200/900	CMST-DTSO USR	6
Scott Reef Lagoon	14°04.58′	121°52.54′	5/02/2008	28/09/2008	5663.1	51	200/900	CMST-DTSO USR	6
Scott Reef SE	14°20.11′	122°09.94′	11/08/2009	2/05/2010	6322.25	339	200/900	CMST-DTSO USR	6
	14°20.13′	122°10.02′	2/05/2010	9/03/2011	7475.75	339	200/900	CMST-DTSO USR	6
Maret Is.	14°24.80′	124°53.66′	14/09/2006	1/03/2007	4037	48	200/900	CMST-DTSO USR	6
	14°24.87′	124°53.64′	1/04/2007	29/11/2007	5794.75	48	200/900	CMST-DTSO USR	6
	14°24.86′	124°53.69′	30/11/2007	11/08/2008	6125.25	48	200/900	CMST-DTSO USR	6
Kimberley IMOS	15°29.00′	121°15.06′	20/11/2012	29/09/2013	7510.25	221	300/900	CMST-DTSO USR	6
	15°29.00′	121°15.06′	1/10/2013	2/06/2014	5852.25	221	300/900	CMST-DTSO USR	6
	15°29.00′	121°15.06′	19/08/2014	8/05/2015	6286.6	221	300/900	CMST-DTSO USR	6
Lacepede Is.	16°50.42′	121°41.56′	13/08/2009	20/04/2010	6015.5	32	200/900	CMST-DTSO USR	6
James Price Point	17°26.72′	122°02.73′	17/06/2009	25/12/2009	4591.75	21	180/900	CMST-DTSO USR	8
CANPASS 1	18°05.05′	120°24.11′	11/09/2017	9/05/2018	5740	102	3600/3600	OBS	0.1
CANPASS 2	17°27.30′	119°57.05′	11/09/2017	9/05/2018	5761	300	3600/3600	OBS	0.1
CANPASS 3	17°06.42′	119°49.12′	11/09/2017	14/06/2018	6623	405	3600/3600	OBS	0.1
CANPASS 4	16°28.89′	119°29.26′	10/09/2017	10/05/2018	5799	853	3600/3600	OBS	0.1
CANPASS 5	16°11.77′	119°17.09′	10/09/2017	10/05/2018	5805	1533	3600/3600	OBS	0.1
CANPASS 6	15°54.52′	119°04.86′	10/09/2017	10/05/2018	5811	2336	3600/3600	OBS	0.1
CANPASS 7	15°28.76′	118°46.30′	9/09/2017	10/05/2018	5847	4162	3600/3600	OBS	0.1
Pilbara region									
Dampier IMOS	19°23.29′	115°54.90′	20/11/2012	27/09/2013	7461.75	224	300/900	CMST-DTSO USR	6
	19°22.51′	115°56.05′	11/08/2014	13/06/2015	7342	223	300/900	CMST-DTSO USR	6
NWS N	19°08.57′	115°13.98′	2/10/2021	3/06/2022	5856	1514	300/1200	LH	96
	19°08.49′	115°13.73′	4/06/2022	17/02/2023	6195.761	1514	400/900	CMST-DTSO USR	6
Montebello NW	19°52.83′	115°14.83′	18/12/2005	21/03/2006	2228.25	234	200/900	CMST-DTSO USR	6
	19°52.83′	115°14.83′	21/03/2006	27/11/2006	6022.5	234	200/900	CMST-DTSO USR	6
	19°51.97′	115°15.86′	6/05/2009	23/07/2009	1863.25	225	200/900	CMST-DTSO USR	6
	19°52.96′	115°16.46′	24/07/2009	23/03/2010	5811.667	192	200/900	CMST-DTSO USR	6
	19°51.85′	115°17.11′	25/03/2010	6/11/2010	5422.25	191	200/900	CMST-DTSO USR	6
Montebello NE	19°56.71′	115°32.64′	17/09/2010	29/03/2011	4626.5	71	200/900	CMST-DTSO USR	6
Montebello	20°11.28′	115°23.57′	24/03/2010	26/10/2010	5183.5	41	200/900	CMST-DTSO USR	6
Barrow Is.	20°36.64′	114°47.89′	17/01/2010	2/09/2010	5469.5	194	200/900	CMST-DTSO USR	6
	20°36.64′	114°47.89′	5/09/2010	9/01/2011	3018.25	194	200/900	CMST-DTSO USR	6
Port Hedland	20°08.32′	118°23.58′	1/11/2011	15/03/2012	3252	14	300/900	CMST-DTSO USR	10
	20°08.32′	118°23.57′	28/03/2012	25/07/2012	2848	14	300/900	CMST-DTSO USR	10
	20°08.36′	118°23.62′	25/07/2012	18/09/2012	1319.5	14	300/900	CMST-DTSO USR	10
Thevenard Is.	21°25.01′	114°50.00′	16/04/2009	20/07/2009	2275.75	39	200/900	CMST-DTSO USR	16
	21°25.00′	114°49.96′	22/07/2009	4/11/2009	2506.5	39	200/900	CMST-DTSO USR	16
	21°25.00′	114°50.00′	14/12/2009	25/03/2010	2423.5	39	200/900	CMST-DTSO USR	6
	21°24.97′	114°49.92′	26/03/2010	4/11/2010	5353.5	40	200/900	CMST-DTSO USR	6
Onslow	21°32.03′	115°01.94′	12/09/2009	22/03/2010	4587.25	11	200/900	CMST-DTSO USR	8
	21°32.01′	115°01.95′	26/03/2010	12/11/2010	5541	11	200/900	CMST-DTSO USR	6
NWS E2	20°17.15′	115°10.76′	30/09/2021	12/05/2022	5376	72	300/1200	LH	96
	20°17.13′	115°10.97′	2/06/2022	31/01/2023	5853.33	70	300/1200	LH	96
NWS E1	20°02.78′	114°53.70′	30/09/2021	2/06/2022	5880	972	300/1200	LH	96
	20°02.77′	114°53.54′	2/06/2022	16/02/2023	6214.51	980	400/900	CMST-DTSO USR	6
NWS C	19°46.84′	114°33.77′	1/10/2021	1/06/2022	5832	1361	300/1200	LH	96
	19°46.75′	114°33.46′	1/06/2022	2/02/2023	5910.67	1364	300/1200	LH	96
	19°46.61′	114°35.13′	31/12/2018	20/03/2019	1886.97	150	400/900	ST500	96
	19°47.47′	114°34.48′	31/12/2018	15/05/2019	3220.75	1300	180/900	WLA	96
	19°48.96′	114°38.46′	15/05/2019	16/08/2019	2232	200	200/900	ST500	96
	19°49.86′	114°38.10′	15/05/2019	11/08/2019	2112	1300	180/900	WLA	96
	19°50.42′	114°35.40′	11/08/2019	31/01/2020	4152	200	200/900	ST500	96
	19°46.66′	114°37.29′	11/08/2019	29/12/2019	3360	1300	180/900	WLA	96
NWS W1	19°23.00′	114°06.72′	1/10/2021	3/06/2022	5880	1154	300/1200	LH	96
	19°22.82′	11°46.41′	3/06/2022	18/02/2023	6235.01	1154	400/900	CMST-DTSO USR	6
NWS W2	18°57.51′	113°37.14′	1/10/2021	3/06/2022	5880	1258	300/1200	LH	96
	18°57.53′	113°37.15′	2/06/2022	4/02/2023	5916.92	1258	300/1200	LH	96
Exmouth Plateau W	19°55.53′	113°13.73′	15/01/2010	18/05/2010	2948.5	939	200/900	CMST-DTSO USR	16
	19°55.53′	113°13.73′	19/05/2010	12/08/2010	2049.75	939	200/900	CMST-DTSO USR	16
Exmouth Plateau	20°23.60′	11°43.89′	6/10/2011	18/01/2012	2497.5	1161	200/900	CMST-DTSO USR	6
	20°28.73′	113°58.02′	29/09/2021	30/09/2021	24	1149	300/1200	LH	96
	20°28.75′	113°57.86′	1/06/2022	14/02/2023	6204.75	1148	400/900	CMST-DTSO USR	6
Gascoyne region									
NW Cape	21°45.08′	113°52.71′	29/04/2019	21/02/2020	7152	358	300/900	CMST-DTSO USR	6
	21°45.08′	113°52.71′	16/05/2018	13/05/2019	8688	358	300/900	CMST-DTSO USR	6
Temperate regions									
Abrolhos Is.	28°45.12′	113°37.02′	20/04/2021	13/11/2021	4967.5	165	900/1800	ST500	48
	28°45.23′	113°37.09′	12/11/2021	15/06/2022	5151.25	161	300/900	CMST-DTSO USR	6
Perth Canyon	31°50.53′	115°00.82′	28/11/2013	4/11/2014	8171.5	391	300/900	CMST-DTSO USR	6
	31°51.77′	115°01.74′	23/09/2016	26/08/2017	8083.25	411	300/900	CMST-DTSO USR	6
	31°52.66′	115°00.66′	17/12/2015	31/12/2016	9113.67	434	300/900	CMST-DTSO USR	6
Kangaroo Is., SA	36°06.82′	135°52.95′	9/12/2014	17/11/2015	8236.75	176	300/900	CMST-DTSO USR	6
	36°07.06′	135°53.61′	17/11/2015	8/11/2016	8577.5	180	300/900	CMST-DTSO USR	6
	36°07.02′	135°53.63′	22/11/2016	4/11/2017	8335.25	172	300/900	CMST-DTSO USR	6
Portland, VIC	38°32.22′	141°14.85′	29/12/2013	26/11/2014	7960.75	157	300/900	CMST-DTSO USR	6
	38°32.52′	141°13.26′	3/02/2015	25/01/2016	8566.75	166	300/900	CMST-DTSO USR	6
	38°32.75′	141°13.27′	29/02/2016	20/02/2017	8567.5	170	300/900	CMST-DTSO USR	6
Tuncurry, NSW	32°19.36′	152°56.67′	10/02/2010	4/10/2010	5664.75	206	500/900	CMST-DTSO USR	6
	32°19.13′	152°56.72′	6/04/2011	26/04/2012	9269.5	207	300/900	CMST-DTSO USR	6
	32°17.40′	152°54.61′	5/06/2012	29/05/2013	8611	136	300/900	CMST-DTSO USR	6
	32°18.59′	152°55.84′	21/02/2016	8/02/2017	8484.25	169	300/900	CMST-DTSO USR	6
Great Barrier Reef region									
Lizard Is.	14°40.86′	145°24.01′	1/12/2014	5/07/2015	5184	26	180/900	CMST-DTSO USR	18

**Table 2:** Percentage of vocal presence hours by month and site. Grey boxes indicate months with no data available.

Location	% Vhr/Month											
	Jan	Feb	Mar	Apr	May	Jun	Jul	Aug	Sep	Oct	Nov	Dec
**Timor Sea**												
Timor Sea					54.87	46.60	54.02	98.96				
Bonaparte Gulf NE	87.10	62.55	85.01	84.88	99.87	70.83	73.92	82.93	71.66	86.38	99.54	95.61
Bonaparte Gulf SW	88.04	60.88	55.53	78.21	87.14	68.43	61.38	56.99	78.21	91.22	98.80	93.82
**Kimberley region**												
Browse Island	39.45	59.79	65.99	25.46	19.56	43.89	20.36	40.14	65.47	56.92	28.11	16.20
Scott Reef N	73.79	15.19				27.04	6.99	4.30	18.19	38.98	15.00	69.09
Scott Reef Lagoon		0	0	0	0	0	0	0	0			
Scott Reef SE	67.27	74.85	93.65	95.83	57.51	41.67	62.63	11.24	59.72	44.83	48.68	59.95
Maret Islands	0.61	0.37	0.13	0.91	0.13	0	0	0	0	0	6.55	3.97
Kimberley IMOS	85.39	57.64	67.07	68.84	33.01	9.38	7.12	3.66	35.61	80.30	75.18	77.69
Lacepede Is.	0.40	1.49	1.88	0.21				0	0	0	0.42	1.21
James Price Pt.						0	0	0.27	0	0	0	0
CANPASS 1	100	98.66	100	99.86	61.27				18.93	70.83	94.44	98.66
CANPASS 2	100	99.55	100	100	74.16				20.65	86.96	99.72	98.12
CANPASS 3	97.85	97.17	97.85	97.78	63.44	49.38			22.01	84.27	97.62	93.68
CANPASS 4	95.97	74.70	10.08	94.03	44.69				4.527	54.97	91.81	85.75
CANPASS 5	31.59	22.47	29.70	14.58	0				0	7.66	32.5	33.47
CANPASS 6	2.42	3.13	3.36	0	0				0	0	9.44	4.16
CANPASS 7	0	0	3.23	0	0				0	0	0	0
**Pilbara region**												
Dampier IMOS	55.71	39.36	87.30	51.32	18.01	3.61	0	0	0	0.40	14.46	37.30
NWS N	50.00	73.39	94.09	58.47	8.06	0.84	0	0	0	22.96	30.97	66.80
Montebello NW	9.27	9.82	13.61	15.97	2.72	1.11	0	0	0	1.344	4.69	0.37
Montebello NE	10.08	25.89	30.44						0	0	0.56	2.69
Montebello			50.56	23.89	3.09	4.31	0	0	0	0		
Barrow Is.	4.88	12.80	18.41	15.14	1.21	0	0	0	0	0	0	4.17
Port Hedland	0	0	0	0	0	0	0	0	0		0	0
Thevenard	0	0	1.11	0.66	0	0	0	0	0	0	0	0
Onslow	0	0	0	0	0	0	0	0	0	0	0	0
NWS E2	12.63	41.77	80.38	48.61	0.34	0.15	0	0	0	0.34	4.65	7.80
NWS E1	38.51	67.21	90.19	37.64	1.34	0	0	0	0	8.60	12.22	46.57
NWS C	17.55	55.18	46.28	16.25	0.90	0	0	0	0	3.54	9.41	35.19
NWS W1	3.16	28.02	8.47	0	0	0	0	0	0	6.31	10.00	15.19
NWS W2	0	0	0	0	0	0	0	0	0	4.15	2.15	2.76
Exmouth Plat. W	0	0	0	0	0	0	0	0		0	0	0
Exmouth Plateau	1.88	90.32				0	0	0	0	0.24	1.32	16.87
**Gascoyne region**												
NW Cape	15.32	38.07	59.01	37.04	6.44	3.40	0	0	0	0	0	0.27
**Temperate regions**												
Abrolhos Is., WA	0	0	0	0	0	0	0	0	0	0	0	0
Perth Canyon, WA	0	0	0	0	0	0	0	0	0	0	0	0
Kangaroo Is., SA	0	0	0	0	0	0	0	0	0	0	0	0
Portland, VIC	0	0	0	0	0	0	0	0	0	0	0	0
Tuncurry, NSW	0	0	0	0	0	0	0	0	0	0	0	0
**GBR, QLD (tentative Omura’s whale presence)**												
Lizard Is.	8.48	0.45	7.26	5.97	5.78	0	0					16.23

## Data Availability

Publicly available datasets analysed in this study are available from the Integrated Marine Observing System (http://imos.org.au/data/; accessed on 1 March 2019). For all other datasets, the raw data is available from the corresponding author on reasonable request.

## References

[1] Wada S., Oishi M., Yamada T.K. (2003). A newly discovered species of living baleen whale. Nature.

[2] Cerchio S., Andrianantenaina B., Lindsay A., Rekdahl M., Andrianarivelo N., Rasoloarijao T. (2015). Omura’s whales (*Balaenoptera omurai*) off northwest Madagascar: Ecology, behaviour and conservation needs. R. Soc. Open Sci..

[3] Sasaki T., Nikaido M., Wada S., Yamada T.K., Cao Y., Hasegawa M., Okada N. (2006). *Balaenoptera omurai* is a newly discovered baleen whale that represents an ancient evolutionary lineage. Mol. Phylogenetics Evol..

[4] Cerchio S., Yamada T.K., Brownell R.L. (2019). Global distribution of Omura’s whales (*Balaenoptera omurai*) and assessment of range-wide threats. Front. Mar. Sci..

[5] Corkeron P.J., Connor R.C. (1999). Why do baleen whales migrate?. Mar. Mammal Sci..

[6] Geijer C.K., di Sciara G.N., Panigada S. (2016). Mysticete migration revisited: Are Mediterranean fin whales an anomaly?. Mammal Rev..

[7] Rosel P.E., Wilcox L.A., Yamada T.K., Mullin K.D. (2021). A new species of baleen whale (*Balaenoptera*) from the Gulf of Mexico, with a review of its geographic distribution. Mar. Mammal Sci..

[8] Rosel P.E., Wilcox L.A. (2014). Genetic evidence reveals a unique lineage of Bryde’s whales in the northern Gulf of Mexico. Endanger. Species Res..

[9] Best P. (2001). Distribution and population separation of Bryde’s whale *Balaenoptera edeni* off southern Africa. Mar. Ecol. Prog. Ser..

[10] Cerchio S., Andrianantenaina B., Zerbini A., Pendleton D., Rasoloarijao T., Cholewiak D. (2018). Residency, Feeding Ecology, Local Movements and Potential Isolation of the Madagascar Omura’s Whale (*Balaenoptera omurai*) Population; Paper Submitted for Consideration by the IWC Scientific Committee. SC/67b/NH/09. https://archive.iwc.int/pages/download.php?direct=1&noattach=true&ref=9468&ext=pdf&k=.

[11] Sousa A.G., Harris D. (2015). Description and seasonal detection of two potential whale calls recorded in the Indian Ocean. J. Acoust. Soc. Am..

[12] Leroy E.C., Royer J.-Y., Alling A., Maslen B., Rogers T.L. (2021). Multiple pygmy blue whale acoustic populations in the Indian Ocean: Whale song identifies a possible new population. Sci. Rep..

[13] McPherson C., Kowarski K., Delarue J., Whitt C., MacDonnell J., Martin B. (2016). Passive Acoustic Monitoring of Ambient Noise and Marine Mammals—Barossa Field: JASCO Document 00997, Version 1.0.

[14] McCauley R. (2011). Woodside Kimberley Sea Noise Logger Program, Sept-2006 to June-2009: Whales, Fish and Man-made Noise: Report R2010-50_3.

[15] Yamada T.K., Kemper C., Tajima Y., Umetani A., Janetzki H., Pemberton D. (2006). Marine mammal collections in Australia. Natl. Sci. Mus. Monogr..

[16] Erbe C., Dunlop R., Jenner K.C.S., Jenner M.-N.M., McCauley R.D., Parnum I., Parsons M., Rogers T., Salgado-Kent C. (2017). Review of underwater and in-air sounds emitted by Australian and Antarctic marine mammals. Acoust. Aust..

[17] Ottewell K., Coughran D., Gall M., Irvine L., Byrne M. (2016). A Recent Stranding of Omua’s Whale (*Balaenoptera omurai*) in Western Australia. Aquat. Mamm..

[18] Samaran F., Stafford K.M., Branch T.A., Gedamke J., Royer J.-Y., Dziak R.P., Guinet C. (2013). Seasonal and geographic variation of southern blue whale subspecies in the Indian Ocean. PLoS ONE.

[19] Mellinger D., Stafford K., Moore S., Dziak R., Matsumoto H. (2007). An overview of fixed passive acoustic observation methods for cetaceans. Oceanography.

[20] Erbe C. (2013). Underwater passive acoustic monitoring & noise impacts on marine fauna—A workshop report. Acoust. Aust..

[21] Mellinger D., Barlow J. Future directions for acoustic marine mammal surveys: Stock assessment and habitat use. Proceedings of the Contribution (Pacific Marine Environmental Laboratory (U.S.)).

[22] Mooney T.A., Di Iorio L., Lammers M., Lin T.-H., Nedelec S.L., Parsons M., Radford C., Urban E., Stanley J. (2020). Listening forward: Approaching marine biodiversity assessments using acoustic methods. R. Soc. Open Sci..

[23] Praca E., Gannier A., Das K., Laran S. (2009). Modelling the habitat suitability of cetaceans: Example of the sperm whale in the northwestern Mediterranean Sea. Deep. Sea Res. Part I Oceanogr. Res. Pap..

[24] Smith J.N., Grantham H.S., Gales N., Double M.C., Noad M.J., Paton D. (2012). Identification of humpback whale breeding and calving habitat in the Great Barrier Reef. Mar. Ecol. Prog. Ser..

[25] Kerosky S.M., Širović A., Roche L.K., Baumann-Pickering S., Wiggins S.M., Hildebrand J.A. (2012). Bryde’s whale seasonal range expansion and increasing presence in the Southern California Bight from 2000 to 2010. Deep. Sea Res. Part I Oceanogr. Res. Pap..

[26] Soldevilla M.S., Debich A.J., Garrison L.P., Hildebrand J.A., Wiggins S.M. (2022). Rice’s whales in the northwestern Gulf of Mexico: Call variation and occurrence beyond the known core habitat. Endanger. Species Res..

[27] Rosel P.E., Corkeron P.J., Engleby L., Epperson D.M., Mullin K. (2016). Status Review of Bryde’s Whales (Balaenoptera Edeni) in the Gulf of Mexico under the Endangered Species Act. NOAA technical memorandum NMFS-SEFSC.

[28] Moreira S.C., Weksler M., Sousa-Lima R.S., Maia M., Sukhovich A., Royer J.-Y., Marcondes M.C.C., Cerchio S. (2020). Occurrence of Omura’s whale, *Balaenoptera omurai* (Cetacea: Balaenopteridae), in the Equatorial Atlantic Ocean based on passive acoustic monitoring. J. Mammal..

[29] Herman L.M. (2017). The multiple functions of male song within the humpback whale (*Megaptera novaeangliae*) mating system: Review, evaluation, and synthesis. Biol. Rev..

[30] Cerchio S., Dorning S., Andrianantenaina B., Cholewiak D.M. (2017). A first description of rhythmic song in Omura’s whale (*Balaenoptera omurai*). J. Acoust. Soc. Am..

[31] Cerchio S., Clark C.W., Garland E.C. (2022). The Omura’s whale: Exploring the enigma. Ethology and Behavioral Ecology of Mysticetes.

[32] McDonald M.A., Mesnick S.L., Hildebrand J.A. (2006). Biogeographic characterization of blue whale song worldwide: Using song to identify populations. J. Cetacean Res. Manag..

[33] Cholewiak D.M., Cerchio S., Jacobsen J.K., Urbán-R J., Clark C.W. (2018). Songbird dynamics under the sea: Acoustic interactions between humpback whales suggest song mediates male interactions. R. Soc. Open Sci..

[34] IMOS (2023). Acoustic Observatories. https://imos.org.au/facility/national-mooring-network/acoustic-observatories.

[35] McCauley R.D., Thomas F., Parsons M.J.G., Erbe C., Cato D.H., Duncan A.J., Gavrilov A.N., Parnum I.M., Salgado-Kent C.P. (2017). Developing an Underwater Sound Recorder: The Long and Short (Time) of It. Acoust. Aust..

[36] Gürlap (2024). Ocean Bottom Seismometers. https://www.guralp.com/products/ocean-bottom-seismometers.

[37] LHI (2024). LS Recorders. https://www.loggerhead.com/ls1-ls2-recorders.

[38] OI (2024). SoundTrap ST500 STD–Long Term Recorder. https://www.oceaninstruments.co.nz/product/soundtrap-st500-std/.

[39] WLA (2017). Song Meter SM3M Submersible and Deep Water. https://www.wildlifeacoustics.com/uploads/user-guides/SM3M-USER-GUIDE.pdf.

[40] Aviation N. (2021). We Had Another Visit Today from Some Omura’s Whales. We Saw This Mother and Calf and also Another Adult That. Facebook [Photographs]. https://www.facebook.com/permalink.php?story_fbid=pfbid0jgxHuBb4BgcyZG8NSPzE8LEvBkTefubfoMpKpfZx4WtUWoF5qmb3Bsurkk6vtQvBl&id=958722754197366.

[41] Aviation N. (2018). Mystery Whales. Yesterday We Saw Four Feeding Whales, Each around 15 Metres Long. Still Trying to Identify What Type They Were. Facebook. https://www.facebook.com/permalink.php?story_fbid=1664475753622059&id=958722754197366.

[42] Sutton A.L., Jenner K.C.S., Jenner M.-N.M. (2019). Habitat associations of cetaceans and seabirds in the tropical eastern Indian Ocean. Deep. Sea Res. Part II Top. Stud. Oceanogr..

[43] Double M.C., Andrews-Goff V., Jenner K.C.S., Jenner M.-N., Laverick S.M., Branch T.A., Gales N.J. (2014). Migratory movements of pygmy blue whales (*Balaenoptera* musculus brevicauda) between Australia and Indonesia as revealed by satellite telemetry. PLoS ONE.

[44] DoE (2015). Conservation Management Plan for the Blue Whale–A Recovery Plan under the Environment Protection and Biodiversity Conservation Act 1999.

[45] Aviation N. (2022). We Saw This Cool and Not So Common Fin Whale out on Tour Today with @Whalesharkdive. The Whale Hung Around. Facebook. https://www.facebook.com/permalink.php?story_fbid=pfbid03n8sySE4TpigcMw34DP7W63EHZoYpT5nxDjHx1UP1gvh2HJbqKmURReTSTkYSXZsl&id=958722754197366.

[46] Aviation N. (2023). A Big Blue at a Banquet. We Found This Pygmy Blue Whale Circling around Scooping up as much as It Could Hold. Facebook. https://www.facebook.com/photo.php?fbid=723938143068342&set=pb.100063564884107.-2207520000.&type=3.

[47] Thums M., Ferreira L.C., Jenner C., Jenner M., Harris D., Davenport A., Andrews-Goff V., Double M., Möller L., Attard C.R. (2022). Pygmy blue whale movement, distribution and important areas in the Eastern Indian Ocean. Glob. Ecol. Conserv..

[48] Clarke R. (2011). Wildlife Images: Omura’s Whale. https://www.pbase.com/wildlifeimages/omuras_whale.

[49] Erbe C., Peel D., Smith J.N., Schoeman R.P. (2021). Marine acoustic zones of Australia. J. Mar. Sci. Eng..

[50] Forrest T., Miller G., Zagar J. (1993). Sound propagation in shallow water: Implications for acoustic communication by aquatic animals. Bioacoustics.

[51] Stafford K.M., Mellinger D.K., Moore S.E., Fox C.G. (2007). Seasonal variability and detection range modeling of baleen whale calls in the Gulf of Alaska, 1999–2002. J. Acoust. Soc. Am..

[52] Erbe C., Verma A., McCauley R., Gavrilov A., Parnum I. (2015). The marine soundscape of the Perth Canyon. Prog. Oceanogr..

[53] Hildebrand J.A. (2009). Anthropogenic and natural sources of ambient noise in the ocean. Mar. Ecol. Prog. Ser..

[54] Thode A.M., D’Spain G., Kuperman W. (2000). Matched-field processing, geoacoustic inversion, and source signature recovery of blue whale vocalizations. J. Acoust. Soc. Am..

[55] Lewis L.A., Calambokidis J., Stimpert A.K., Fahlbusch J., Friedlaender A.S., McKenna M.F., Mesnick S.L., Oleson E.M., Southall B.L., Szesciorka A.R. (2018). Context-dependent variability in blue whale acoustic behaviour. R. Soc. Open Sci..

[56] Stimpert A.K., DeRuiter S.L., Falcone A.E., Joseph J., Douglas A.B., Moretti D.J., Friedlaender A.S., Calambokidis J., Gailey G., Tyack P.L. (2015). Sound production and associated behavior of tagged fin whales (*Balaenoptera physalus*) in the Southern California Bight. Anim. Biotelemetry.

[57] Thode A., Bonnel J., Thieury M., Fagan A., Verlinden C., Wright D., Berchok C., Crance J. (2017). Using nonlinear time warping to estimate North Pacific right whale calling depths in the Bering Sea. J. Acoust. Soc. Am..

[58] Cato D.H., Noad M.J., McCauley R.D. (2005). Passive Acoustics as a Key to the Study of Marine Animals, in Sounds in the Sea: From Ocean Acoustics to Acoustical Oceanography.

[59] GBRMPA (2016). Sighting Wildlife–13103 Other or Unidentified (Whales). https://eotr.gbrmpa.gov.au/sightings/sightingwildlife/13103.

[60] GBRMPA (2016). Sighting Wildlife–13236 Other or Unidentified (Whales). https://eotr.gbrmpa.gov.au/sightings/sightingwildlife/13236.

[61] Cooke J.G., Brownell R. *Balaenoptera omurai* (Amended Version of 2018 Assessment). The IUCN Red List of Threatened Species 2019: E.T136623A144790120.

[62] Department of the Environment 2024 Balaenoptera omurai in Species Profile and Threats Database, Department of the Environment, Canberra. https://www.environment.gov.au/sprat..

[63] Evenden C. (2023). Oil and Gas Titles Australia.

[64] Erbe C., Dunlop R., Dolman S. (2018). Effects of noise on marine mammals. Effects of Anthropogenic Noise on Animals.

[65] Thomas P.O., Reeves R., Brownell R.L. (2016). Status of the world’s baleen whales. Mar. Mammal Sci..

[66] Williams R., Wright A., Ashe E., Blight L., Bruintjes R., Canessa R., Clark C., Cullis-Suzuki S., Dakin D., Erbe C. (2015). Impacts of anthropogenic noise on marine life: Publication patterns, new discoveries, and future directions in research and management. Ocean Coast. Manag..

[67] DoEWHA (2008). EPBC Act Policy Statement 2.1–Interaction between Offshore Seismic Exploration and Whales 2008.

